# An Audit of Compliance With NICE Guideline: Obesity: Identification, Assessment and Intervention by the Forensic Community Mental Health Team Based at a Supported Accommodation

**DOI:** 10.1192/bjo.2022.492

**Published:** 2022-06-20

**Authors:** Chung Mun Alice Lin, Neeti Sud

**Affiliations:** 1 National Institute of Health Research Newcastle Biomedical Research Centre and the Translational and Clinical Research Institute, Newcastle Upon Tyne, United Kingdom; 2 Cumbria, Northumberland, Tyne and Wear NHS Foundation Trust, Newcastle Upon Tyne, United Kingdom

## Abstract

**Aims:**

In England, 64.8% of adults are currently classified as overweight or obese, with rates even higher in the North East at 68.6%, especially in adults with severe mental health illnesses. This additional body weight has the potential to increase the risk of developing a number of serious health conditions including diabetes, heart disease and even cancer. Studies have shown that patients with schizophrenia have a 2.8–3.5 increased likelihood of significant weight gain and reduction in life expectancy of 15–20 years, mainly due to preventable physical illness. Monitoring of risk factors for this, particularly weight gain, is therefore crucial. The NICE Guideline (2014) recommends that patients are routinely categorised into BMI categories to assist with obesity identification, management, and monitoring. A waist measurement is also advised to help with risk stratification. Patients with psychosis or schizophrenia, especially those taking anti-psychotics are also recommended to be offered a combined healthy eating and physical activity programme by their mental healthcare provider. Finally, patients with rapid or excessive weight gain, abnormal lipid levels or problems with blood glucose management should be offered appropriate interventions. Our main objective was to identify whether the obesity assessment, monitoring and intervention care delivered by our community team is in line with current guidance.

**Methods:**

A total of 12 residents living in community forensic supported accommodation and currently taking antipsychotic medications were included. Data reviewed were from September 2020 to September 2021. Data audited were from electronic medical records.

**Results:**

This audit found that 10 out of 12 patients (83%) fell into either the overweight or obese BMI categories (seven obese and three overweight). Only four patients had agreed to have their waist circumference measured, which meant only four patients were able to be appropriately risk stratified. One patient was identified as pre-diabetic and another diabetic. All patients identified to be overweight or obese received appropriate lifestyle advice. Qrisk scores, to assess cardiovascular risk, were calculated for the majority of eligible patients, except for two.

**Conclusion:**

This audit highlights that patients who are on regular antipsychotic treatment and living in the community are at high risk of obesity and its associated complications. It is important to perform regular health checks in this cohort due to this risk, both to improve their quality of life and prevent significant morbidity and mortality. Waist circumference measurements should be encouraged to enable risk stratification and accurate documentation will enable timely treatment intensification.

